# Workflow for Harmonic IR and Raman Spectra of Embedded Systems: The PE-QM Approach

**DOI:** 10.1021/acs.jpca.5c00713

**Published:** 2025-07-18

**Authors:** Jonas Vester, David Carrasco-Busturia, Kenneth Ruud, Magnus Ringholm, Jógvan Magnus Haugaard Olsen

**Affiliations:** † DTU Chemistry, 5205 Technical University of Denmark (DTU), DK-2800 Kongens Lyngby, Denmark; ‡ Division of Theoretical Chemistry and Biology, School of Engineering Sciences in Chemistry, Biotechnology and Health, 530019 KTH Royal Institute of Technology, SE-100 44 Stockholm, Sweden; § Hylleraas Centre for Quantum Molecular Sciences, Department of Chemistry, 8016 UiT The Arctic University of Norway, N-9037 Tromso̷, Norway

## Abstract

We present a workflow, benchmarks, and applications to provide a roadmap for simulating harmonic IR and Raman spectra for solute–solvent systems by employing a polarizable-embedding quantum-mechanics (PE-QM) approach. This multiscale modeling scheme divides the system into a central core region described by quantum-mechanical methods and an environment region described through the fragment-based polarizable embedding (PE) model. The workflow involves generating representative structures, calculating properties, and postprocessing data. Benchmark calculations quantify errors introduced by some of the key approximations used in our approach and discuss its strengths and weaknesses. Finally, we apply the workflow to acetone in three different solvents, comparing simulated spectra to experimental results to further evaluate our approach and identify potential weaknesses. Accurate simulations of solute–solvent systems are an important step toward modeling more complex molecular systems with a fragment-based PE approach.

## Graphical abstract

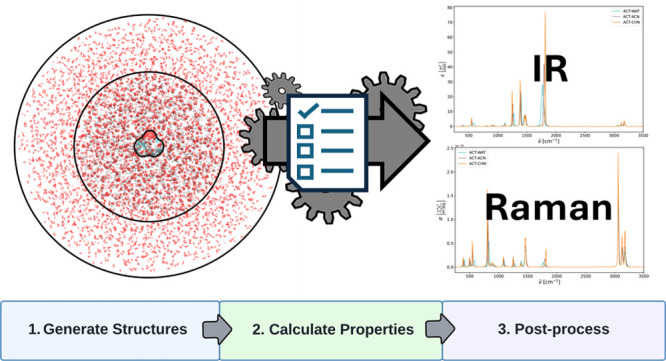

## 1 Introduction

The study of molecular vibrations, as probed by vibrational spectroscopy, provides a unique lens through which the intricacies of chemical systems can be unraveled. However, the complexity of molecular systems poses challenges in interpreting experimental vibrational spectra. Large molecular systems often contain thousands of overlapping signals, each corresponding to a different molecular vibration. In such cases, theoretical research and computational studies can complement experimental studies as interpretative tools at the molecular scale. Yet, the computational demands increase substantially for large molecular systems, ranging from thousands to potentially millions or even billions of atoms. This is especially true when quantum mechanics is essential, such as in simulations of chemical reactions and light-matter interactions. Existing computational methods based on molecular quantum mechanics face limitations, particularly when considering thermal effects. To address this challenge and significantly reduce computational costs, a multiscale quantum mechanics/molecular mechanics (QM/MM) approach is often employed.^1–5^ In this approach, the system is divided into a quantum core region modeled using quantum mechanics (QM) and a classical environment region described by an atomistic molecular mechanics (MM) force field. This is important for capturing anisotropic interactions, such as hydrogen bonds, between the two regions. For vibrational properties, these calculations provide invaluable insights into the chemical structure, bonding characteristics, and intermolecular interactions of these systems.^5–8^

If a static approach is used, i.e., where the atom positions are fixed in the property calculation, the environment description can be reduced to only include parameters that directly affect the core region. This can be referred to as a focused approach, as it only considers the full details of the core region and how it is affected by the environment. One such approach is the fragment-based polarizable embedding model (PE)^9,10^ that will be the focus of this work. In Figure 1, we demonstrate the application of it to a solute–solvent system, highlighting the partitioning into the core and environment regions. Various polarized embedding models, including the fragment-based PE model, have been applied for calculating analytical vibrational properties for different vibrational spectroscopies.^11–16^

**Figure 1:**
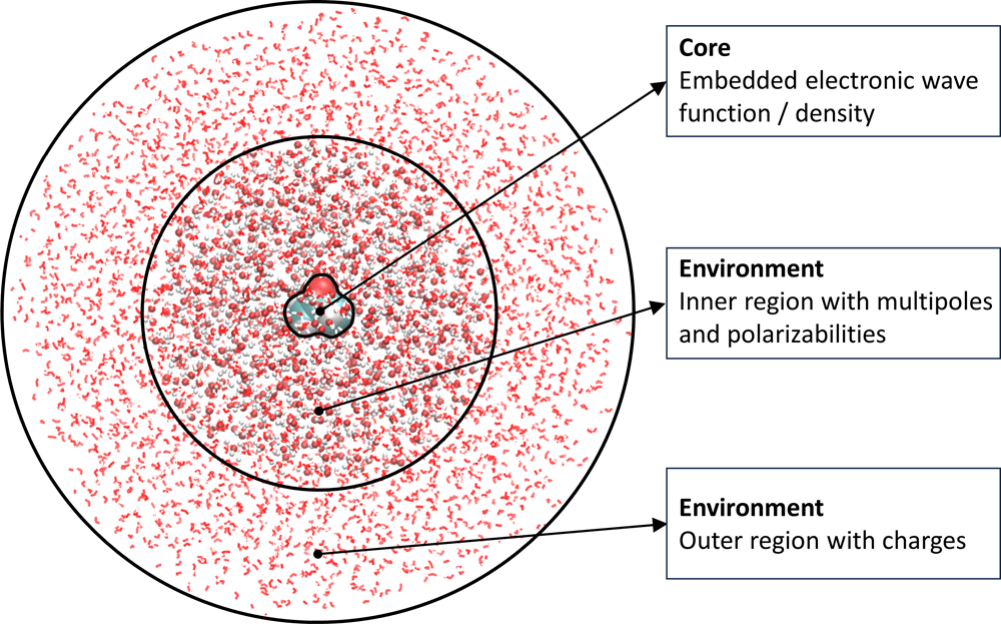
Illustration of the multiscale approach used in this work. The solute–solvent system is partitioned into a core region and an environment region that is further subdivided into an inner and outer region. The core contains the solute molecule, while solvent molecules are part of the inner or outer regions or removed depending on the distance from the core region.

The static approach aligns naturally with the partial Hessian vibrational analysis (PHVA) approximation,^17–19^ which focuses on selected portions of the Hessian matrix of the entire system. PHVA has been effectively applied in QM/MM studies^20–22^ to evaluate vibrational properties. Here, only the Hessian of the core region is used, while the off-diagonal blocks between the core and environment are disregarded, along with the diagonal block exclusive to the environment. In our previous work, we implemented PHVA in a PE-QM framework to enable the modeling of harmonic IR and Raman spectra for solute–solvent systems.^14^

To streamline the approach, we present in this work a comprehensive workflow detailing key simulation steps. Benchmark calculations, using the acetone in water solute–solvent system, are used to assess the accuracy of various approximations employed in different parts of the workflow. As part of the benchmarks, we investigate the effects of pseudotranslational and pseudorotational contributions, i.e., vibrational motions that resemble translational or rotational motions of the core region, relying on tools provided in our previous work,^23^ in which we investigated the PHVA approximation in QM/MM-based vibrational analysis. Additionally, we simulate harmonic IR and Raman spectra for acetone in other solvents, comparing our results with experimental data to evaluate the performance of the approach and identify potential weaknesses that need to be addressed. The clear understanding of the reliability and limitations of our methodology obtained in this work is a key step on the path from the simpler solute–solvent systems to the modeling of more complex biomolecular systems, such as lipid membranes, proteins, and nucleic acids.

## 2 Methodology

### 2.1 Computational Workflow

The workflow for simulating harmonic IR and Raman spectra of solute–solvent systems is shown in Figure 2. It is divided into three main steps, each encompassing three tasks, and is based on the commonly used sequential QM/MM (S-QM/MM) approach.^24^ In this approach, molecular mechanics simulations are used to efficiently sample a large ensemble of configurations, which are then analyzed using quantum mechanical or QM/MM calculations on selected subsets to obtain statistically converged averages of the target properties. The workflow presented here builds on the S-QM/MM framework to address vibrational properties, and a similar methodology has been explored by the group of Cappelli^25^ based on QM/FQ.^26^ Both approaches share common elements, such as the use of QM/MM methods to analyze configurations generated via molecular mechanics sampling, the incorporation of a polarizable environment, and the focus on calculating localized properties using quantum mechanics. However, they differ in some aspects, such as the description and partitioning of the polarizable environment. While both methods are, in principle, generally applicable to any molecular system, QM/FQ’s application has mainly been limited to a few solvents.^27^ One study has explored a chromophore embedded in a protein environment;^28^ however, that work highlights that parametrization remains a critical challenge for applying this method to large biological systems. In contrast, the fragment-based PE approach can be readily applied to any solvent and molecular system that can be fragmented into computationally manageable fragments. The workflow presented in the following refines and adapts the S-QM/MM approach with a specific focus on harmonic IR and Raman spectra, providing a tailored framework for these applications.

**Figure 2:**
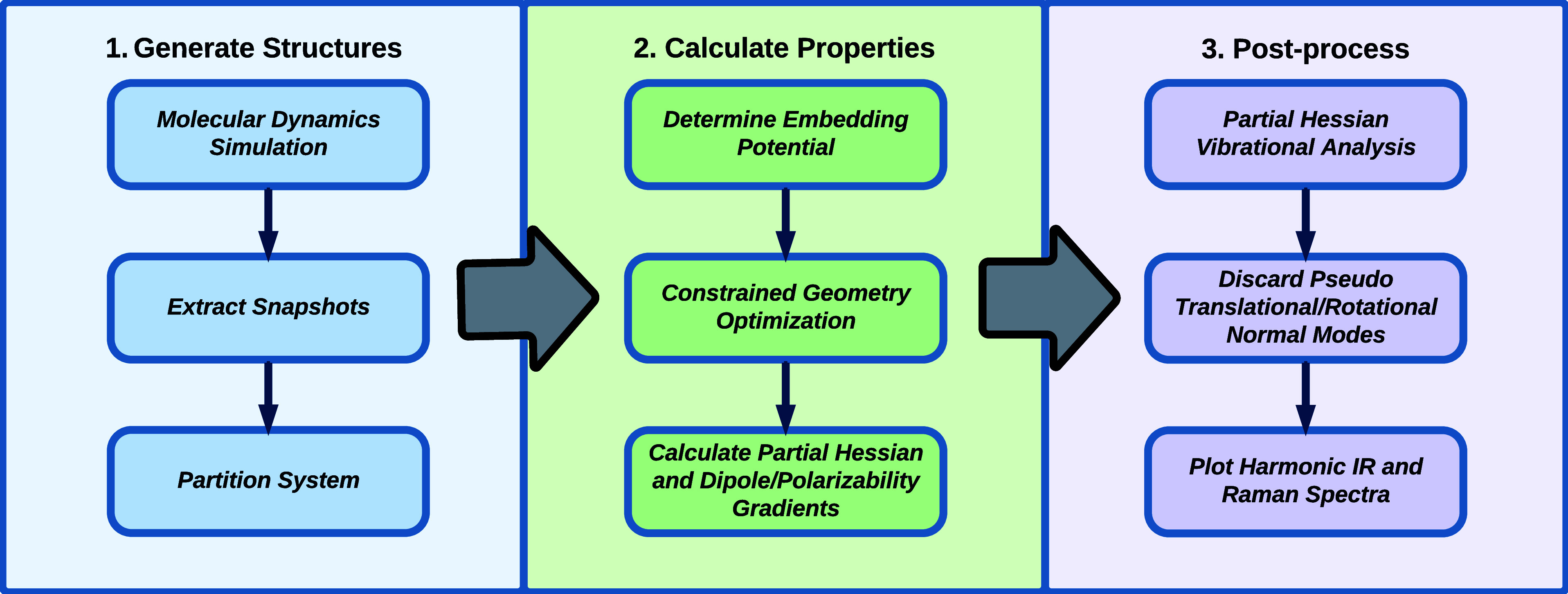
Computational workflow for the simulation of harmonic IR and Raman spectra of solute–solvent systems.

The first major step in this workflow is to generate a suitable ensemble of structures. The configurational sampling is usually done by running a molecular dynamics (MD) simulation. From the resulting trajectory, a number of snapshots with a specific time separation are extracted. To determine the minimum number of snapshots and the optimal time step between them for obtaining converged spectra, it is necessary to benchmark the sample size. This involves examining how the convergence of frequencies and intensities calculated for the ensemble of snapshots depends on different step sizes between snapshots and varying total numbers of snapshots. After extracting a suitable number of structures, they are partitioned into a core subsystem and an environment subsystem. The environment can be further subdivided into different subsystems, e.g., into an inner polarizable part and an outer nonpolarizable part, as illustrated in Figure 1, to reduce the computational cost. For this step, the size of the molecular environment must be benchmarked to ensure that the vibrational frequencies and intensities are converged with respect to the system size.

The second major step is calculating the relevant properties for the set of structures produced in the previous step. This process begins by fragmenting the environment subsystem, or its inner part, into smaller, computationally manageable fragments. Next, the embedding parameters for each fragment are determined and assembled to produce the complete embedding potential. The parameters for the outer part, if present, can be taken from a nonpolarizable force field. For solvents, the fragments simply correspond to individual solvent molecules. In contrast, environments containing large molecules, such as proteins, require a more sophisticated fragmentation approach.^29–31^ For each fragment, atom-centered multipoles and polarizabilities are calculated. This methodology has been shown to produce highly accurate embedding potentials across a wide range of systems and properties.^30,32–40^ Then, a constrained geometry optimization is performed in which the geometry of the core region is optimized while keeping the environmental atoms fixed. This preserves, to some degree, the temperature effects in the environment and its interaction with the core subsystem. The partial Hessian, partial dipole gradients, and partial polarizability gradients, all of which are derivatives with respect to the positions of the nuclei in the core region, are then calculated based on the geometry-optimized structures.

The third and final major step is the postprocessing of the properties calculated in step two. The partial Hessian is mass-weighted and diagonalized to yield the vibrational frequencies and the corresponding normal modes. The dipole and polarizability gradients are mass-weighted, transformed using the eigenvectors from the diagonalization of the Hessian, and used to calculate the IR and Raman intensities, respectively. Then, normal modes that include vibrational motions between the core region and the environment, which look like translational or rotational motions of the core region, are discarded since they are not well described by the model. We refer to these vibrational motions as pseudotranslations and pseudorotations. Finally, the harmonic IR and Raman spectra are plotted using the vibrational frequencies and intensities of all structures convoluted with an appropriate broadening function.

### 2.2 Computational Details

This study considers one solute and three solvents: acetone in water, acetone in cyclohexane, and acetone in acetonitrile. We selected acetone as the solute for our model system due to its simple vibrational spectrum and semipolar characteristics, which enable it to dissolve in both polar and nonpolar solvents. The solvents were selected based on their varying polarities: water is polar, cyclohexane is nonpolar, and acetonitrile is intermediate in polarity.

The molecular structures were generated through classical MD simulations. The acetone, cyclohexane, and acetonitrile molecules were modeled using an improved version of the General AMBER Force Field, GAFF2.^41,42^ The topologies were generated by first optimizing the geometry of the molecules at the HF/6–31G* level of theory using Q-Chem.^43^ Then the geometries are passed to ACPYPE,^44^ a Python script that uses Antechamber^42,45^ and facilitates the generation of molecular topologies. Water molecules were described with TIP3P.^46^ All three cubic simulation boxes were 60 Å in length, and the number of water, cyclohexane, and acetonitrile molecules was fixed to 7145, 1203, and 2489 molecules, so that the densities of the solvents at 300 K were reproduced (0.997, 0.779, and 0.786 g/cm^3^). For acetone in water, the gmx solvate functionality^47^ was used, while Packmol^48^ was used for the other two systems. All MD simulations were performed using GROMACS 2019.4.^47^ First, an energy minimization using the steepest descent algorithm with a maximum force tolerance of 1000 kJ mol^–1^ nm^–1^ and a maximum of 50,000 steps was performed. This was followed by an equilibration run of 100 ps and a production run of 20 ns using a time step of 1 fs. The velocity rescaling thermostat^49^ with 0.1 ps time constant was used to maintain a temperature of 300 K. The short-range van der Waals, and Coulomb electrostatic cutoff distances were both set to 1.0 nm. Particle mesh Ewald^50^ was used for fast calculation of long-range electrostatic interactions, and SETTLE^51^ constraints in rigid TIP3P^46^ water were applied.

Using the geometries from the MD simulation, the multiscale modeling approach shown in Figure 1 was applied. The effects from the solvent were modeled by embedding potentials produced using PyFraME.^52^ For the geometry optimizations, the geomeTRIC package^53^ has been employed together with the LSDalton program package^54,55^ and the FraME library.^56^ The calculated molecular properties were the Hessian matrix, the dipole gradients, and the polarizability gradients at an incident wavelength of 514.5 nm. These properties were calculated using LSDalton with the OpenRSP library,^57–59^ where the additional environment contributions were also included using FraME.

The benchmark calculations (Section 3.1) were carried out at the HF/pcseg-1 ^60^ level of theory for acetone in water. The core region included a single acetone molecule, and the environment was partitioned into two spheres surrounding the acetone molecule containing water molecules. To keep the computational cost low, we chose the inner region to have a radius of 8 Å and the outer region a radius of 30 Å. The standard potential model SEP^36^ was used for the polarizable embedding in the inner region and TIP3P^46^ for the charges in the outer region of the environment. The Lennard-Jones (LJ) 12–6 parameters used for the inner and outer regions were taken from the OPLS-AA force field.^61^

From the MD simulation, 3000 snapshots with a step size of 1.0 ps were extracted starting after 1 ns. During the geometry optimization and calculation of molecular properties, some snapshots were found to have convergence issues or to be statistical outliers. To identify the latter, we calculated the absolute Z-score for each snapshot, which is defined as,

$$Z=\frac{\left|x-\mu \right|}{\sigma }$$ 1

where *Z* is the standard score, *x* is the observed value (in our case, the frequency, IR intensity, and Raman intensity), μ is the sample mean, and σ is the standard deviation of the sample. We discarded all snapshots with a *Z*-score above 5. Removing the discarded snapshots and the snapshots with convergence issues left us with 2890 snapshots, which were then used for the benchmark calculations. From the 110 discarded snapshots, the geometry optimizations for 28 snapshots did not converge. The remaining snapshots, which we consider statistical outliers, display subtle geometric distortions of the acetone molecule, which may correlate with the observed anomalously high vibrational frequencies and intensities using PE-QM, making them unsuitable for inclusion in our representative data set.

To benchmark the sample size, we defined windows of different step sizes and numbers of snapshots, for which we calculated the average frequencies, IR intensities, and Raman intensities. We calculated the average window error δ̅ by first calculating the average frequencies and intensities μ_*i*,*j*_ for each window *i* for each individual normal mode *j*. Then, we calculated the absolute error by subtracting the value of the reference window μ_ref_ for each normal mode of each window:

$$|\mu_{i,j}-\mu_{\mathrm{ref},j}|=\Delta \mu_{i,j}$$ 2

The reference window consists of the total set of snapshots used during the benchmark calculations, that is, the 2890 nonoutliers and converged snapshots obtained with a step size of 1 ps, and is assumed to be converged with respect to sampling the configurational space. Then, we averaged over all normal modes *j* to get the average error per window $\overset{-}{\Delta \mu_{i}}$ with respect to frequencies, IR intensities, and Raman intensities. To further condense the data, we averaged the error per window $\overset{-}{\Delta \mu_{i}}$ for all windows *i* to get the overall average window error δ̅. Additionally, we calculated the corresponding standard deviation and identified the maximum error among all windows when calculating δ̅.

Additionally, we investigated the effect of using different LJ 12–6 parameters on acetone in water for 30 snapshots separated by 2 ps. The settings for this investigation mirrored those described below for the calculations of acetone in water, except for the LJ 12–6 parameters. Specifically, we tested the TIP3P,^46^ TIP3P-CHARMM,^62^ and SPC/E^63^ parameters to simulate the harmonic IR and Raman spectra. Furthermore, we calculated the harmonic IR and Raman spectra using TIP3P with a nonpolarizable inner region, employing electrostatic embedding in place of the polarizable embedding used previously. During the geometry optimizations where the SPC/E and TIP3P parameters were utilized, one snapshot each failed to converge. Consequently, only 29 snapshots were used for these cases.

The application of our workflow to solvent shifts in acetone (Section 3.2) is carried out at the PBE0 ^64–66^ /pcseg-2 ^60^ level of theory for all three solute–solvent systems. We chose this functional due to its accuracy in the modeling of molecular geometries,^65^ and the basis set because it has been shown to yield good results with DFT for both molecular structures and vibrational properties.^67^ The grid used was a standard type grid in LSDalton called GRID5, which has a radial integration accuracy of 2.15443 × 10^–17^ and an angular expansion order of 47. The inner region had a radius of 16 Å (this choice is based on the benchmark calculations in Section 3.1), and the outer region had a radius of 30 Å for all three solute–solvent systems. For the inner region, atom-centered multipoles up to quadrupoles and atom-centered dipole–dipole polarizabilities were derived using the LoProp scheme,^68,69^ based on properties calculated with the Dalton program package^55,70^ employing PBE0 and a recontracted version of the 6-31+G* basis set named loprop-6-31+G* in Dalton.^71–73^ The generation of the embedding potentials was facilitated using PyFraME.^52^ The LJ 12–6 parameters for the core, and inner and outer environment regions, as well as the charges used only for the outer region, were taken from the GAFF2 force field or TIP3P in the case of water.

The IR and Raman intensities were derived from the partial dipole gradients and the partial polarizability gradients, respectively, according to the scheme described by Dundas et al.,^14^ where the IR intensity is reported in terms of the molar decadic attenuation coefficient ϵ and the Raman intensity is related to the absolute differential Raman cross sections σ′.^74^ The frequencies and normal modes were obtained from the eigenanalysis of the partial Hessian.

To plot the IR and Raman spectra, the Cauchy–Lorentz distribution was used for the line shape function with a half-width at half-maximum (HWHM) of 3.0 cm^–1^ for all normal modes. The Cauchy-Lorentz distribution used in this work is

$$f(\overset{-}{\nu };\overset{-}{\nu_{I}},\gamma_{I})=\pi^{-1}\left[\frac{\gamma_{I}}{(\overset{-}{\nu_{I}}-\overset{-}{\nu }{)}^{2}+\gamma_{I}^{2}}\right]$$ 3

where ν̅ is the wavenumber of the incident radiation, ν̅_I_ is the wavenumber associated with vibrational mode I, and γ_I_ is the scale parameter for mode I and specifies the HWHM.

## 3 Results and Discussion

### 3.1 Benchmark Calculations

Benchmark calculations are required to evaluate the accuracy of our approach to calculate harmonic IR and Raman spectra of solute–solvent systems. To this end, we investigated the pseudotranslational and pseudorotational contributions to all normal modes, as well as the convergence with respect to the basis set size, system size, and configurational sample size. Additionally, we briefly examined the effect of choosing different LJ 12–6 parameters obtained from commonly used force fields and explored the role of mutual polarization in PE-QM by comparing those results with electrostatic embedding quantum mechanics (EE-QM). In the following, the 30 normal modes of acetone have been labeled in descending order from 1 (highest frequency) to 30 (lowest frequency).

The partial Hessian eigenanalysis yields 3*N* normal modes, where *N* represents the number of atoms in the core region. At least six of these modes (or five in the case of a linear core) are associated with full or partial pseudotranslational and pseudorotational normal modes. These pseudotranslational and pseudorotational components arise within the PHVA approximation and reflect vibrational motions between atoms in the core and the environment. However, since the environment atoms are fixed, these vibrations appear as translational or rotational motions of the core. In our previous work,^23^ we showed that normal modes with substantial pseudotranslational or pseudorotational contributions produce inaccurate frequencies and intensities compared to their full Hessian counterparts. As a result, these modes should be excluded from further analysis.

To identify pseudotranslation and pseudorotation, we use the absolute square of the dot product between the normalized eigenvectors and normalized vectors corresponding to the translational and rotational motion of the atoms in the core region (in Cartesian coordinates). In Figure 3, we present the average pseudotranslational and pseudorotational contributions across all 2890 snapshots for an inner-sphere size of 8 Å and an outer-sphere size of 30 Å. The results reveal that the eight lowest-frequency modes have substantial pseudotranslational and pseudorotational contributions. The maximum values and standard deviations show that the degree of pseudotranslational and pseudorotational contributions highly depends on the specific configuration. Therefore, it is crucial to consider the entire set of snapshots when identifying pseudotranslational and pseudorotational contributions. Outside of those eight modes, the average values, maximum values, and standard deviations are close to zero. Based on these results, the eighth lowest-frequency normal modes were not included in the following analyses. This exclusion includes low-frequency modes that, in principle, are experimentally observable but cannot be studied using our model due to the limitation of having fixed atoms in the environment.

**Figure 3:**
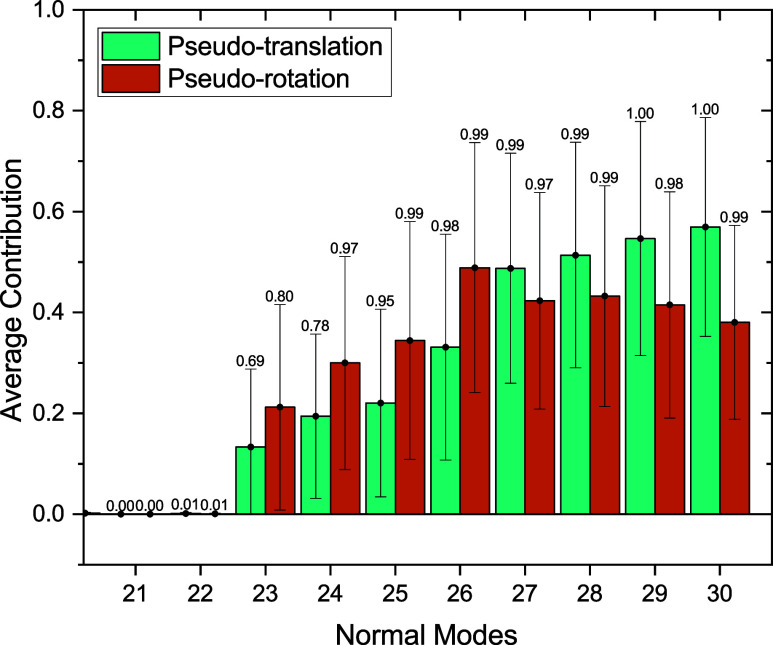
Pseudotranslational and pseudorotational contributions of the lowest-frequency normal modes of acetone in water, averaged over all snapshots. The error bars show the standard deviation, and the values above the bars are maximum values.

Additionally, we examined the influence of different inner-sphere sizes (8–24 Å) on the pseudotranslational and pseudorotational contributions within a single snapshot (see Figure S1 in the Supporting Information). The results show that further increasing the size of the inner sphere has a minor effect on the pseudotranslational and pseudorotational contributions of acetone in water. This observation is anticipated, as only the solvent molecules in close proximity to the core region interact strongly with it. As the next step in refining our understanding of the computational accuracy of our approach, we investigated the impact of basis set size. For this, we employed the segmented contracted versions of the polarization-consistent basis sets (pcseg-*n*).^60^ The convergence of vibrational frequencies, IR intensities, and Raman intensities for a single snapshot of acetone in vacuum is shown in Figures 4 and 5, using pcseg-4 as the reference. A similar analysis for acetone in water was conducted using pcseg-3 as the reference (see Figures S2 and S3 in the Supporting Information). However, we consider the results for acetone in vacuum to be more reliable, as pcseg-4 provides a more accurate reference. Due to performance limitations in our pilot implementation, it was not possible to generate pcseg-4 reference data for acetone in water. Despite this, we observed that the basis set errors for acetone in vacuum and acetone in water were similar when pcseg-3 was used as the reference for both. Therefore, we believe it is reasonable to use acetone in vacuum as the reference for assessing basis set errors in this study.

**Figure 4:**
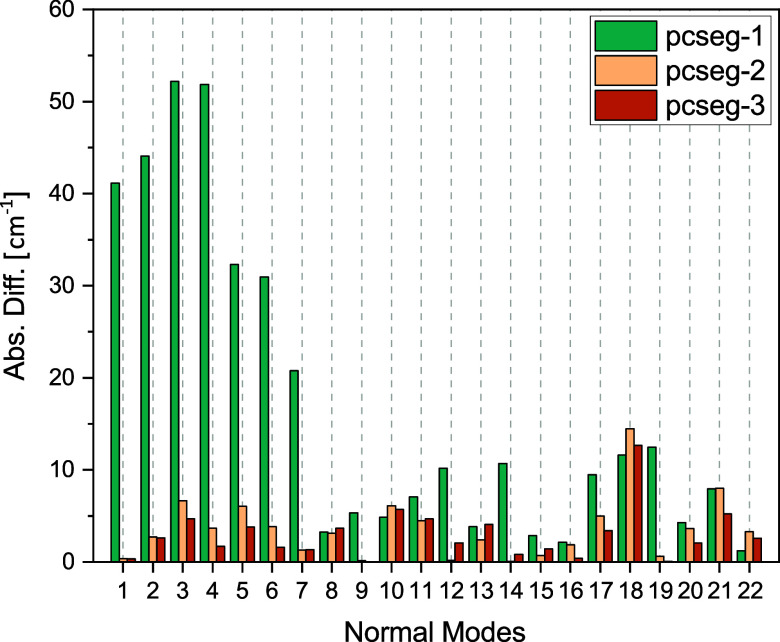
Convergence of vibrational frequencies with respect to basis set sizes for a single snapshot of acetone in vacuum. Shown are absolute differences relative to reference values calculated using pcseg-4 for acetone in vacuum.

**Figure 5:**
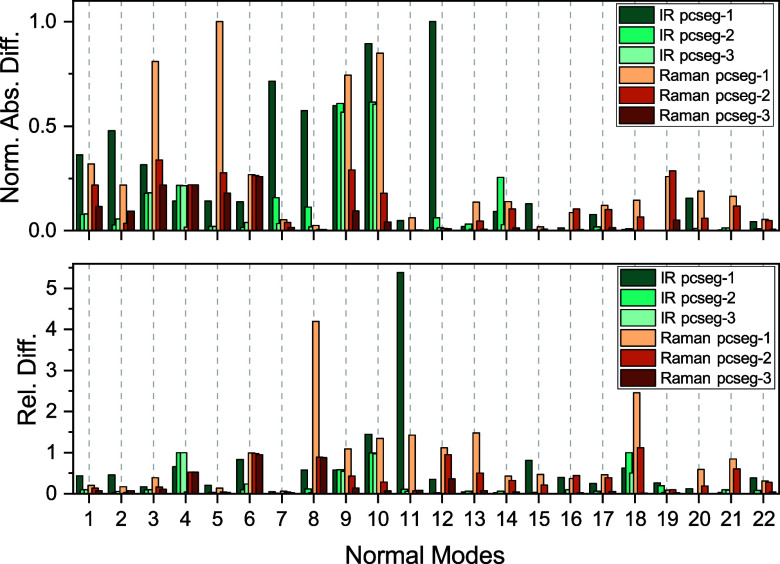
Convergence of IR and Raman intensities with respect to basis set size for acetone in vacuum. Shown are normalized absolute differences and relative differences compared to reference values calculated using pcseg-4. The absolute differences have been normalized to the highest absolute difference which for the IR intensities is 2.21 × 10^12^ m^2^ mol and for the Raman intensities it is 1.98 × 10^–56^ C^4^ s^2^ m^–2^ J^–1^ kg^–1^.

In Figure 4, we present the absolute differences of vibrational frequencies with respect to reference values calculated using pcseg-4 for acetone in vacuum. It is clear that the errors are very large when using pcseg-1, especially for the high-frequency modes. Using pcseg-2 substantially reduces the errors, leading us to conclude that pcseg-2 is an adequate choice when also taking into consideration the computational cost. For pcseg-2, the average error ± standard deviation (maximum value) across all normal modes is 3.58 ± 3.36 (14.47) cm^–1^.

Figure 5 presents the normalized absolute differences and relative differences for the IR and Raman intensities of acetone in vacuum. For a difference to be notable in the spectrum, both the relative and absolute differences must be considerable. The results highlight that increasing the basis set size from pcseg-1 to pcseg-2 reduces intensity errors. However, a few normal modes still exhibit substantial absolute and relative differences. For IR, these include normal modes 4 (normalized absolute difference 0.22, relative difference 1.0), 9 (normalized absolute difference 0.61, relative difference 0.58), and 10 (normalized absolute difference 0.61, relative difference 0.99). For Raman, notable differences are observed in normal modes 6 (normalized absolute difference 0.26, relative difference 0.97) and 9 (normalized absolute difference 0.29, relative difference 0.43). For these specific modes, increasing the basis set size to pcseg-3 does not significantly improve their intensities, except for normal mode 9 in the case of Raman.

The simulation box used for MD simulations of solvated systems is sometimes larger than necessary for embedding calculations of molecular properties. In such cases, the system can be truncated to reduce the computational cost. For solute–solvent systems, the so-called droplet model can be a reasonable approach. In this model, all solvent molecules within a given distance from the core region are included while the remainder are removed. Here, we further partitioned the environment into two regions, the inner and outer regions, as depicted in Figure 1, where the inner region is described through multipoles and polarizabilities, and the outer region through charges only. The main purpose of the outer region is to avoid artificial polarization at the outer boundary of the inner region, potentially allowing for a smaller inner region and thus reducing the computational cost. However, as shown by Kvedaravičiu̅tė et al.,^75^ this approach does not always work, and instead, the minimum-image convention could be used.^75^ In this work, due to a rather inefficient implementation, it is crucial to keep the size of the polarizable part of the environment as small as possible to further reduce the computational costs that arise predominantly during the calculation of the Hessian. We are working on a more efficient implementation, so this is not expected to be an issue in the future.

To investigate the convergence with respect to the size of the polarizable inner region, we used reference values calculated using an inner-sphere radius of 24 Å and kept the outer region at a constant size with a radius of 30 Å in all calculations. In Figure 6, we show the absolute differences of the frequencies with increasing sizes of the inner region. We find that already at 8 Å the maximum errors are below 3 cm^–1^, and they drop to below 1 cm^–1^ at 14 Å. In Figures 7 and 8, we present the normalized absolute and relative differences for the IR and Raman intensities. Here, we see that there is only one case where both the absolute and relative errors are notable at the same time, namely the Raman intensity of normal mode 11 when using an inner-sphere radius of 8 Å. However, by increasing the sphere size, the errors go down substantially. Since the overall intensity errors are small and to keep the maximum errors well below 1 cm^–1^, we chose a 16 Å radius for the inner sphere. For this particular choice, the average error ± the standard deviation (maximum error) is 0.14 ± 0.24 (0.79) cm^–1^.

**Figure 6:**
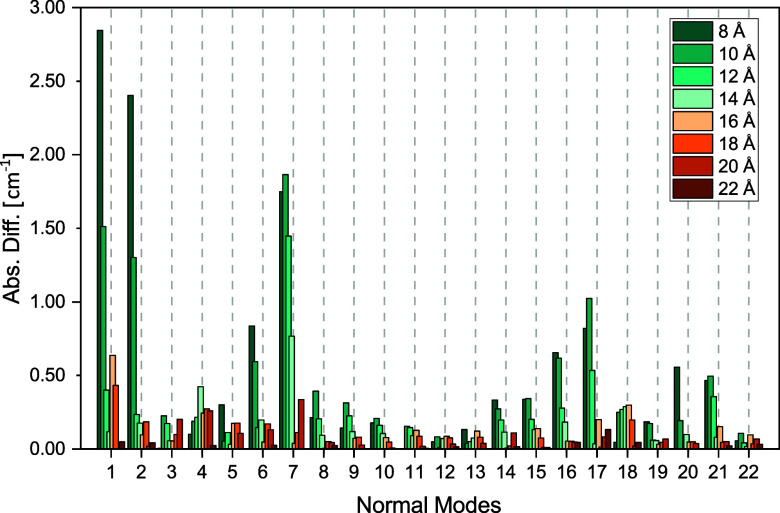
Convergence of vibrational frequencies with respect to the size of the polarizable inner region for a single snapshot of acetone in water. The outer-sphere radius was kept constant at 30 Å. Shown are the absolute differences compared to reference values obtained using an inner-sphere radius of 24 Å and outer-sphere radius of 30 Å.

**Figure 7:**
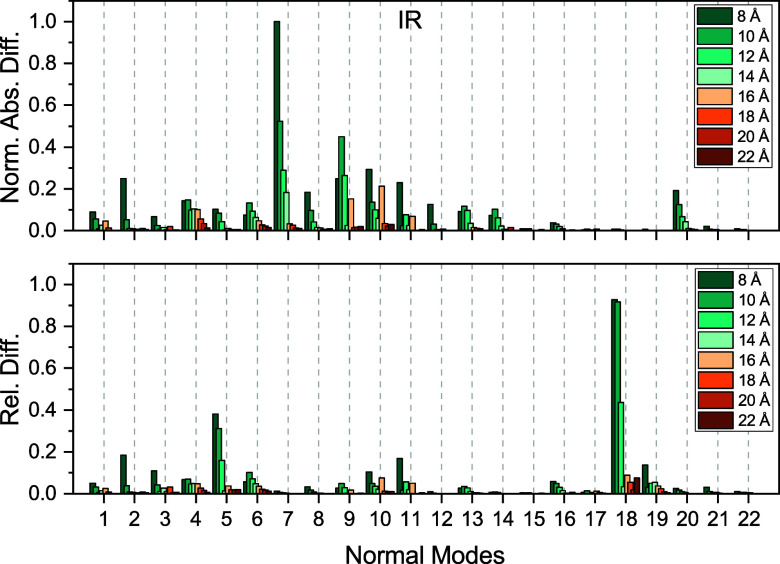
Convergence of IR intensities with respect to the size of the polarizable inner region for a single snapshot of acetone in water. The outer-sphere radius was kept constant at 30 Å. Shown are the normalized absolute differences and relative differences compared to reference values obtained using an inner-sphere radius of 24 Å and outer-sphere radius of 30 Å.

**Figure 8:**
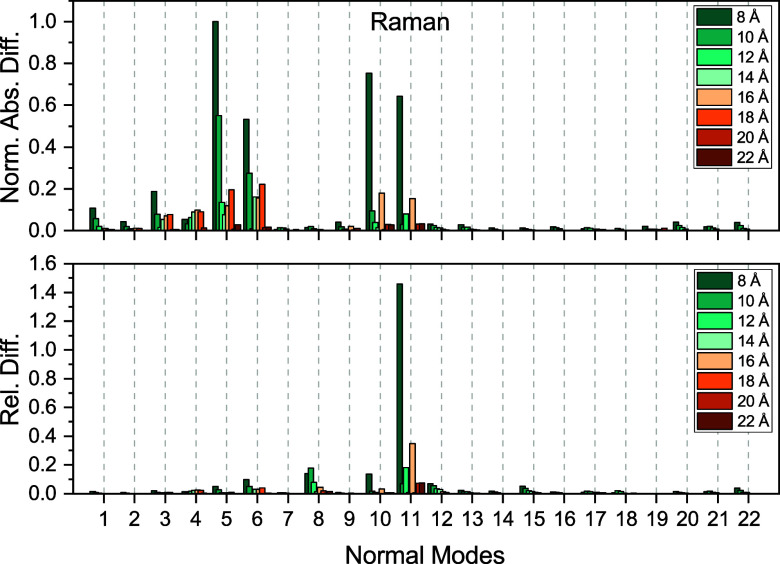
Convergence of Raman intensities with respect to the size of the polarizable inner region for a single snapshot of acetone in water. The outer-sphere radius was kept constant at 30 Å. Shown are the normalized absolute differences and relative differences compared to reference values obtained using an inner-sphere radius of 24 Å and outer-sphere radius of 30 Å.

To adequately capture the dynamics of the system, the sample size is of high importance. When extracting snapshots from the MD trajectory, there are two parameters to consider: the time or step size between each snapshot and the total number of snapshots, which together give the length of the trajectory. The step size should be chosen so as to make it large enough to only extract statistically uncorrelated snapshots. In some cases, it would also be important to choose the smallest possible step size while still adhering to the aforementioned criterion, to reduce the simulation time, e.g., for computationally expensive QM/MM MD simulations. Here, we are using classical MD simulations that are comparatively low in computational cost.

The total number of snapshots defines the largest sample or window that serves as the reference for benchmarking smaller samples. The convergence with respect to the number of snapshots and step size must be assessed simultaneously since the quantities depend on each other. Basing our initial guess for the step size on the hydrogen-bond switching dynamics in liquid water, which has a hydrogen bond lifetime of about 0.78 ps,^76^ led us to investigate step sizes of 1.0, 2.0, 3.0, 4.0, and 5.0 ps for different numbers of snapshots. We found that these step sizes had a minor impact on the results for a given number of snapshots, leading to the average window error mainly depending on the number of snapshots. Therefore, we only present the results for the 2.0 ps step size, while the rest of the data can be found in Tables S1–S4 in the Supporting Information.

The basic assumption in the calibration of the step size and the number of snapshots is that the reference window of 3.0 ns with a step size of 1.0 ps, giving a total of 2890 snapshots, is converged for both quantities. For each different number of snapshots (25, 50, 125, 250, and 500), a set of smaller subwindows, each representing a subset of the data of the reference window, was considered. The subwindow length (step size times the number of snapshots) was shifted by 1.0 ps relative to each other until the end of the reference window was reached. In each subwindow, only snapshots spaced by the step size, starting from the first snapshot, were considered. For each set of subwindows representing a specific step size and number of snapshots, the results were compared to the results of the reference window, leading to an average window error as described in the computational details in Section 3. In other words, the average window error indicates how much, on average, a randomly chosen subwindow of the trajectory (with a given step size and number of snapshots) would deviate from the reference window, which we assume to be converged.

In Table 1, we present the average window errors, standard deviations, and maximum errors regarding frequency, absolute and relative IR intensity, and absolute and relative Raman intensity for 25, 50, 125, 250, and 500 snapshots at a constant 2.0 ps step size. The table also includes the number of windows included in the averages.

**Table 1:** Configurational Sample Size Errors for the Frequencies, IR Intensities, and Raman Intensities^a^

	number of snapshots^b^				
property^c^	500	250	125	50	25
δ̅ν̃	0.2 ± 0.0 (0.3)	0.3 ± 0.1 (0.5)	0.4 ± 0.1 (0.8)	0.7 ± 0.2 (1.6)	1.0 ± 0.3 (2.5)
δ̅*I*_IR_	2.5 ± 0.7 (4.0)	3.2 ± 0.8 (5.0)	4.4 ± 1.1 (9.4)	7.3 ± 2.4 (15.1)	10.7 ± 3.3 (22.4)
δ̅_*r*_ *I*_IR_	1.4 ± 0.5 (2.6)	2.0 ± 0.5 (3.2)	2.9 ± 0.7 (5.3)	4.6 ± 1.3 (9.0)	6.6 ± 1.7 (12.2)
δ̅*I*_Raman_	1.8 ± 0.8 (3.8)	2.5 ± 1.0 (5.3)	3.3 ± 1.3 (7.1)	4.6 ± 1.8 (10.5)	6.3 ± 2.3 (15.1)
δ̅_*r*_ *I*_Raman_	1.1 ± 0.4 (2.0)	1.4 ± 0.4 (2.4)	1.9 ± 0.6 (3.7)	2.7 ± 1.0 (6.0)	3.8 ± 1.2 (9.1)
*N*_windows_ ^d^	945	1195	1320	1395	1420

a Entries are average absolute (δ̅) or relative (δ̅_*r*_) window errors ± standard deviations with maximum errors in parentheses.

b Snapshots are separated by 2.0 ps.

c Frequency errors are in cm^–1^, absolute IR intensity errors are in 10^10^·m^2^ ·mol, absolute Raman intensity errors are in 10^–58^ C^4^ ·s^2^ ·m^–2^ ·J^–1^ ·kg^–1^, and all relative values are percentages.

d Number of windows included in the averages.

The average window error for the frequencies is lowest for 500 snapshots with 0.2 cm^–1^ and gradually increases up to 1.0 cm^–1^ for 25 snapshots, while the standard deviation increases from 0.0 to 0.3 cm^–1^. Additionally, the maximum error is below 1.0 cm^–1^ for 500, 250, and 125 snapshots and only increases to 2.5 cm^–1^ for 25 snapshots. This indicates that the error in the frequencies due to insufficient sampling can be kept quite low with as few as 25 snapshots, i.e., on the order of the basis-set incompleteness error. Similarly, the absolute and relative IR and Raman intensity errors increase gradually when decreasing the number of snapshots from 500 to 25. The relative IR intensity errors range from an average error of 1.4% and a maximum error of 2.6% to an average error of 6.6% and a maximum error of 12.2%. In comparison, the relative Raman intensity errors range from an average error of 1.1% and a maximum error of 2.0% to an average error of 3.8% and a maximum error of 9.1%. To ensure a maximum relative error below 5% for the intensities and the maximum frequency error below 1.0 cm^–1^, we chose 250 snapshots with a 2.0 ps step size to be appropriate for our calculations in Section 3.2.

In summary, our benchmark calculations show that using an inner-sphere radius of 16 Å and a sample size of 250 snapshots with a 2 ps step size introduces an error that is minor compared to the basis-set incompleteness error. We estimate that the overall average error for the frequencies is approximately 4 cm^–1^, with a maximum error of 16 cm^–1^.

Additionally, a brief investigation into the effects of the Lennard-Jones 12–6 parameters and the role of mutual polarization in PE-QM on the harmonic IR and Raman spectra is illustrated in Figure 9, 10, 11, and 12. In addition to the spectra, Tables S5–S7 in the Supporting Information present the absolute differences in frequencies, as well as the absolute and relative differences in harmonic IR and Raman intensities, using PE-QM with LJ (TIP3P) as reference for the different parameter choices. Both the Raman and IR spectra (Figure 9 and 11) reveal that for the different LJ 12–6 parameters with a polarizable environment the differences are minor. The frequency difference is for all modes close to or below 1 cm^–1^, and the relative intensity changes are below 10% for all peaks of visibly strong intensity in the spectra. The comparison between IR and Raman spectra in which the environment is not polarizable and polarizable (Figures 10 and 12) reveals substantial differences, especially for the carbonyl stretching mode ν­(C=O), with a frequency difference of more than 22 cm^–1^ and relative differences of 19% for IR and 17% for Raman intensities. Although more subtle, other noticeable changes are in the low- to midfrequency part of the spectra revealed by the difference spectra.

**Figure 9:**
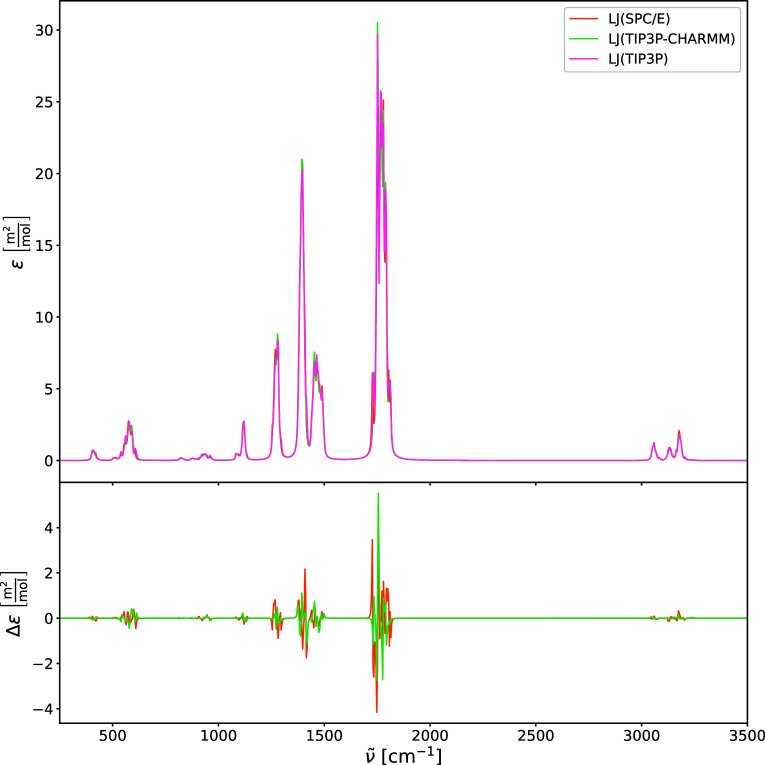
Harmonic IR spectra of acetone in water (upper panel) calculated using PE-QM with different LJ 12–6 parameters: TIP3P, SPC/E, and TIP3P-CHARMM models. The difference spectra (lower panel) are shown relative to TIP3P for comparison.

**Figure 10:**
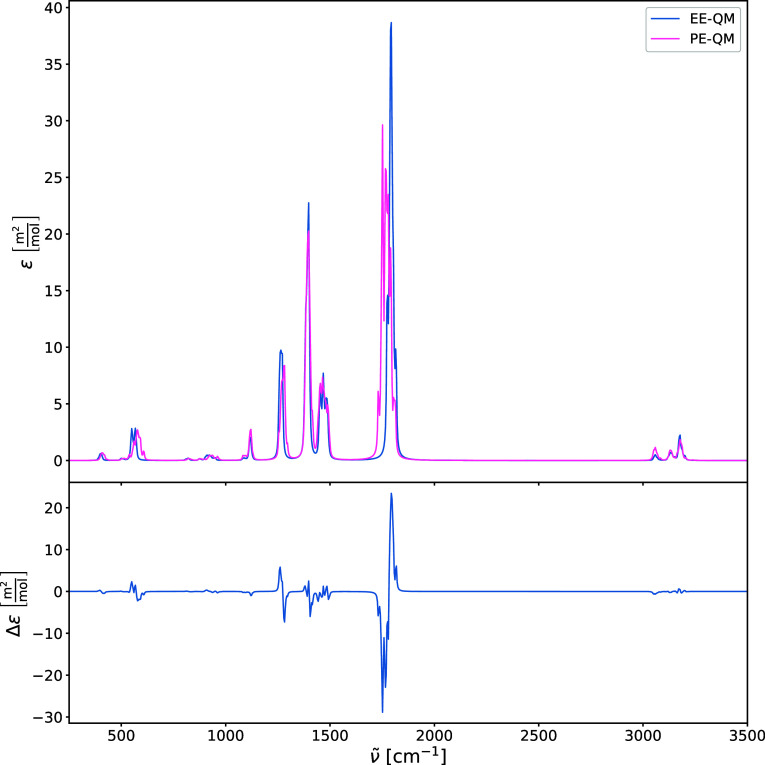
Harmonic IR spectra of acetone in water (upper panel) calculated using PE-QM and electrostatic embedding quantum mechanics (EE-QM). The difference spectra (lower panel) is shown for comparison.

**Figure 11:**
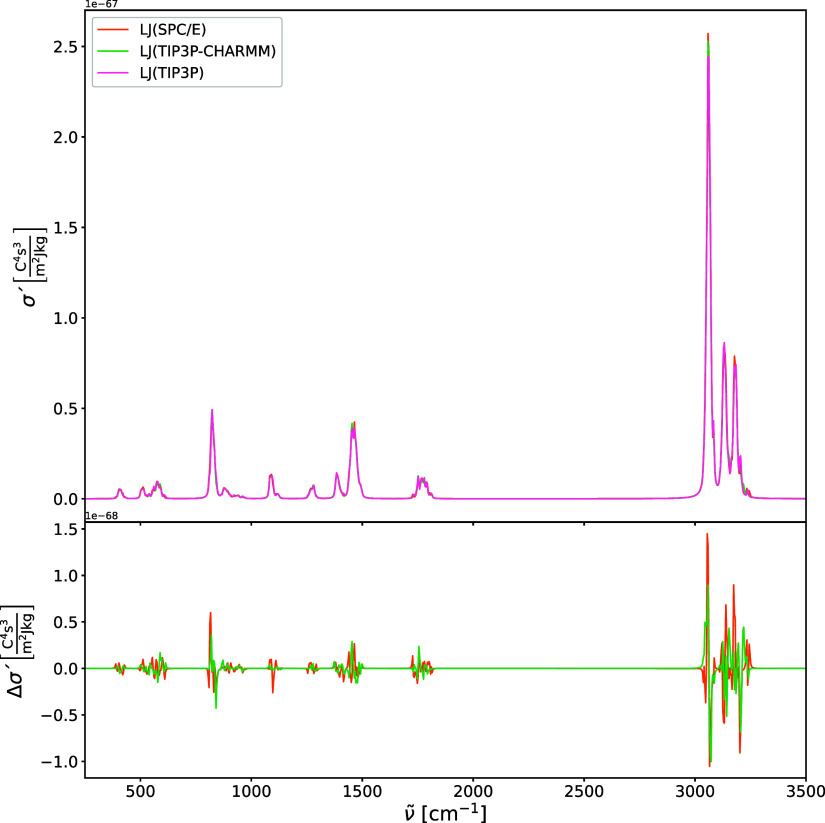
Harmonic Raman spectra of acetone in water (upper panel) calculated using PE-QM with different LJ 12–6 parameters: TIP3P, SPC/E, and TIP3P-CHARMM models. The difference spectra (lower panel) are shown relative to TIP3P for comparison.

**Figure 12:**
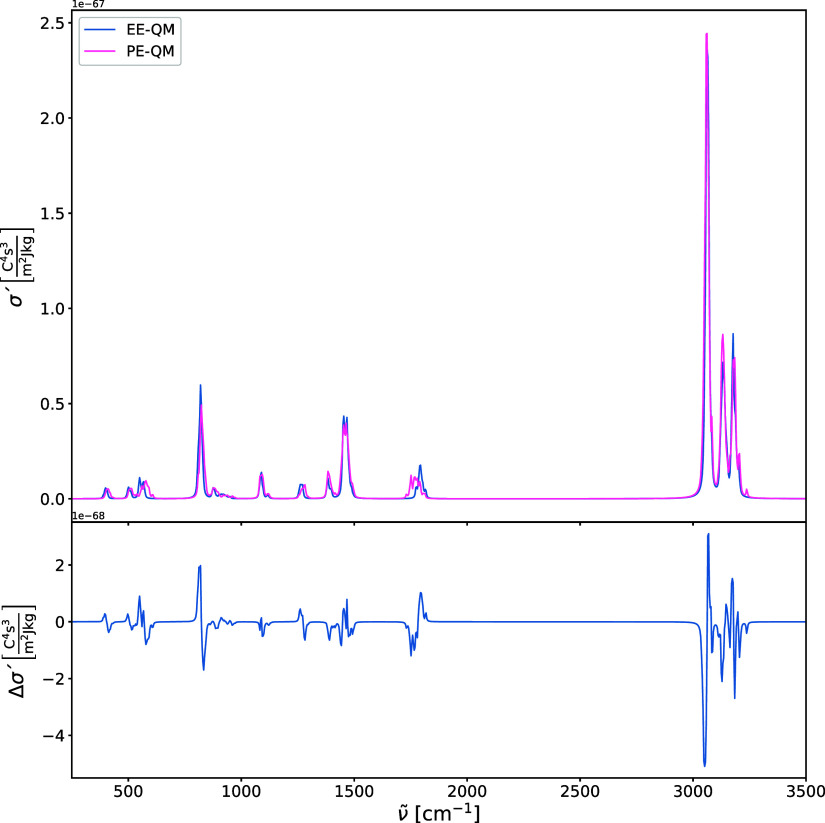
Harmonic Raman spectra of acetone in water (upper panel) calculated using PE-QM and electrostatic embedding quantum mechanics (EE-QM). The difference spectra (lower panel) is shown for comparison.

### 3.2 Application to Solvent Shifts in Acetone

Using the workflow and the results from the benchmark calculations, we have calculated the harmonic IR and Raman spectra for acetone in water, acetone in acetonitrile, and acetone in cyclohexane. Even though the benchmark calculations have only been performed for acetone in water, we assume that of the three systems simulated, water, being the most polar solvent, is the most complex and difficult to simulate of the three systems. Therefore, it is reasonable to assume that the computational parameters obtained from the benchmark results are sufficient and might even be stricter than necessary for the other two solvents.

The number of snapshots we aimed for was 250; however, since some snapshots tend to have convergence issues or are statistical outliers, we started with 260 snapshots. This led to the final number of snapshots used being 249 for acetone in water, 253 for acetone in acetonitrile, and 243 for acetone in cyclohexane. The spectra were convoluted with the line shape function in eq 3, and are presented in Figures 13 and 14. The IR intensities are given by the molar decadic attenuation coefficient ε, while the Raman intensities are given by the absolute differential scattering cross-section σ′.

**Figure 13:**
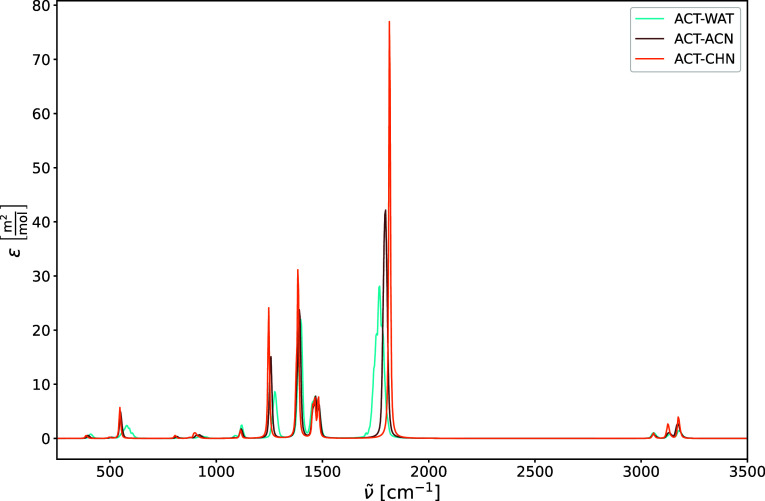
Harmonic IR spectra of acetone in water (ACT-WAT), acetone in acetonitrile (ACT-ACN), and acetone in cyclohexane (ACT-CHN). The IR intensities are expressed in terms of the molar decadic attenuation coefficient ε.

**Figure 14:**
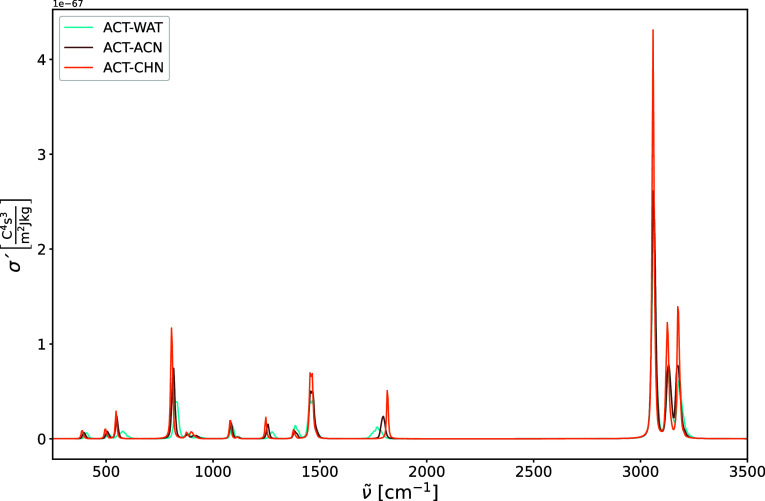
Harmonic Raman spectra of acetone in water (ACT-WAT), acetone in acetonitrile (ACT-ACN), and acetone in cyclohexane (ACT-CHN). The Raman intensities are expressed in terms of the absolute differential scattering cross-section σ′.

The strongest peak in the three IR spectra in Figure 13 can be assigned to the carbonyl stretching mode ν­(C=O) (around 1800 cm^–1^). We limit the following discussion of the IR spectra to this vibrational mode and compare the solvent shifts of the maximum of the carbonyl stretching mode to the experimental results by Nyquist et al.^77^ (see Table 2). Although Nyquist et al. measured the carbonyl stretching frequency for hexane, our simulation used cyclohexane, which has very similar solvent properties. Therefore, we expect the shifts to be comparable. We also assume that the density of the acetone vapor in the experiment was low enough to make it comparable to acetone in vacuum.

**Table 2:** Vibrational Frequencies and Solvent Shifts (in cm^–1^) of the Carbonyl Stretching Mode of Acetone in Various Solvents

solvents	this work	experiment^77^	Δν̃(C=O)
vacuum	1824	1735^a^	89
cyclohexane	1815	1722^b^	93
acetonitrile	1797	1713	84
water	1769	1699	70

solvent shifts			Δshift
cyclohexane – acetonitrile	18	9^b^	9
cyclohexane – water	46	23^b^	23
acetonitrile – water	28	14	14

a Experiment value is for acetone vapor.

b Experiment value is for hexane solvent.

We reproduce the general trend that the frequency of the carbonyl stretching mode is red-shifted and broadened the more polar the solvent. The frequency of the carbonyl stretching mode drops from 1815 cm^–1^ in cyclohexane (nonpolar), to 1797 cm^–1^ in acetonitrile (intermediate polarity), and to 1769 cm^–1^ in water (high polarity). Comparing the solvent shifts, the shift from cyclohexane to acetonitrile is 18 cm^–1^, while the experiment reports a shift of 9 cm^–1^. We thus overestimate the solvent shift by a factor of 2. The same applies to the shift when going from acetonitrile to water, which is 28 cm^–1^ in our work and 14 cm^–1^ experimentally. Using a vibrational scaling factor would reduce these discrepancies. However, the appropriate scaling factor in this case would have a minor impact on the solvent shifts. Our calculated solvent shifts are twice as large as the experimental ones. This result is higher than the maximum basis-set error found in the benchmark calculations (see Sec. 3.1). This can possibly be explained by the errors introduced by the partitioning of our system into a core and environment region. In particular, the hydrogen bonds, which are especially important for the carbonyl stretching mode, cross the two regions and are only partially accounted for by the PE-QM approach.

The most intense peaks in the Raman spectrum are the symmetric C–H stretching modes, whose peaks for all three solvents strongly overlap, suggesting no significant differences in the solute–solvent structure and dynamics. To further investigate the accuracy of our approach, we compare the calculated Raman spectrum of acetone in water to an experimental Raman spectrum (see Figure 15). The calculated spectrum is scaled by a factor of 0.96 which is appropriate for PBE0 and a triple-ζ basis.^78^ Notably, the experimental spectrum contains signals from both acetone and water, while our theoretical simulation only contains the signals from acetone. This explains the left shoulder of the carbonyl stretching mode in the experimental spectrum that comes from the bending mode in water and the large signal above 3000 cm^–1^ that belongs to the symmetric O–H stretching mode in water, which overlays the two highest-frequency C–H stretching modes of acetone. Also, the stronger signal on the lower frequency end is an artifact of the baseline correction applied in the analysis of the experimental spectrum.

**Figure 15:**
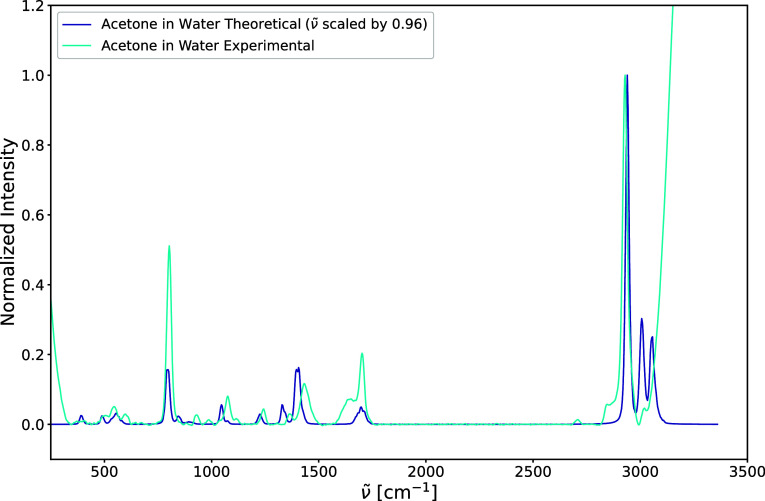
Theoretical harmonic Raman spectrum of acetone in water (ACT-WAT) and experimental Raman spectrum of a 0.5% acetone in water solution (measured at 532 nm over 5 s averaged over 10 accumulations). The baseline of the experimental spectrum has been corrected with the asymmetrically reweighted penalized least-squares smoothing algorithm (arPLS).^80^ The theoretical intensities have been normalized so that the highest intensity of the experimental and theoretical peaks agree. The experimental spectrum has been provided by Martin Maier (Universität Heidelberg, Germany).

The comparison between the theoretical and experimental spectra reveals that the most prominent peaks are present in both. While there are many similarities, some differences in intensity and frequency for certain peaks are observed. The intensity of the C–C stretching mode is more than half as intense in the simulation as in the experiment. The carbonyl stretching mode is also much less intense than in the experiment; however, in the experiment, this mode overlaps with the water bending mode, which makes it difficult to compare directly. Furthermore, the positions of the peaks between 1000 and 1500 cm^–1^, corresponding to the asymmetric methyl deformation modes, deviate by around 25 cm^–1^. This may be due to the need to include anharmonicity, as scaling factors alone may not suffice. It is well-known that normal modes exhibit varying degrees of anharmonicity across different frequency regions. For example, the work of Ringholm et al.^79^ demonstrates that anharmonic frequency corrections derived from analytic cubic and quartic force constants vary across different frequency regions.

## 4 Summary and Outlook

In this work, we presented a computational workflow, benchmark calculations, and applications of our PE-QM scheme to calculate harmonic IR and Raman spectra. The benchmark calculations were performed for acetone in water and the applications for acetone in water, acetone in acetonitrile, and acetone in cyclohexane.

The workflow covers steps essential for generating structures, calculating properties, and postprocessing the properties to the final IR and Raman spectra. This includes configurational sampling through an MD simulation, calculating analytically the partial Hessian and the dipole and polarizability gradients, which are then postprocessed to obtain frequencies and IR and Raman intensities for the different configurations.

We benchmarked our approach for the acetone in water system to evaluate its accuracy. We showed how the pseudotranslational and pseudorotational contributions substantially affect the eight lowest-frequency normal modes that thus should be removed. Additionally, our analysis with regards to basis-set incompleteness, found the basis-set error not to exceed 14.5 cm^–1^. In contrast, the analysis of the size of the polarizable part of the environment and the configurational sample size revealed that the frequency errors can be kept below 1 cm^–1^ at a reasonable computational cost. Adding up the average errors for the frequencies, their standard deviations, and the maximum error for the choices made in our workflows, leads to 4.0 ± 3.7 (15.8) cm^–1^. The basis-set errors in the intensities were quite large for acetone in vacuum, indicating a major impact on the spectra even with pcseg-3 when compared to pcseg-4. The errors in intensities introduced by limiting the size of the polarizable part of the environment and the sample size were, in general, rather small. Even when only considering a polarizable solvent sphere with a radius of 8 Å, the deviations remained minor. Likewise, using just 25 snapshots had little additional impact on the computed intensities. The choice of different LJ 12–6 parameters from commonly used force fields had a small impact. However, using a nonpolarizable description of the environment substantially affected the results, particularly the carbonyl stretching mode, highlighting the importance of mutual polarization in the environment.

Building on our benchmark calculations, we calculated the harmonic IR and Raman spectra of acetone in three different solvents. We used the PBE0/pcseg-2 level of theory with a polarizable solvent sphere of 16 Å radius and a nonpolarizable outer sphere of 30 Å radius. Approximately 250 snapshots were used with a step size of 2.0 ps between them. The eight lowest-frequency modes were removed based on the pseudotranslational and pseudorotational analysis. We compared the solvent shifts of the carbonyl stretching mode to the experimental ones, revealing errors ranging from 9 to 23 cm^–1^. Additionally, we compared our simulated Raman spectrum for acetone in water to an experimental spectrum, displaying many similarities between the spectra. The largest frequency deviations, about 25 cm^–1^, were observed between 1000 and 1500 cm^–1^. The largest deviations in Raman intensities were observed for the C–C stretching mode at around 830 cm^–1^ and the carbonyl stretching mode at around 1769 cm^–1^.

In summary, through our benchmarked workflow, we provide guidelines for how to use a focused PE-QM approach to simulate harmonic IR and Raman spectra of solute–solvent systems. The benchmark calculations quantify the errors introduced through various boundary conditions imposed by our scheme, such as pseudotranslational and pseudorotational contributions, basis-set size, system size, configurational sample size, and choice of LJ 12–6 parameters. Lastly, we compared our theoretical spectra to experimental data, revealing more in-depth strengths and weaknesses. Some aspects warrant further attention. One is the size of the core region when partitioning the system. In this regard, it is unclear what the impact is from the removal of temperature effects and by how much the accuracy can be improved. Of particular interest is how including different types of hydrogen bonds between the core region and the environment region will affect the calculated results.

While our current implementation is limited to small solute–solvent systems, we envision that further optimization through parallelization and algorithmic improvements will allow the methodology to be extended to larger systems.

## Supplementary Material



## Notes

The data set containing all the data and scripts used in this article is available at 10.5281/zenodo.12793099. The Supporting Information is available free of charge at https://pubs.acs.org/doi/10.1021/acs.jpca.5c00713.

Pseudotranslational and pseudorotational contributions of a single snapshot of acetone in water for different polarizable inner-region sizes; convergence of vibrational frequencies and harmonic IR and Raman intensities with respect to basis set sizes for acetone in water, using pcseg-3 as a reference; absolute and relative average window error for step sizes of 1.0, 3.0, 4.0, and 5.0 ps with respect to frequencies, IR intensities, and Raman intensities; absolute frequency differences and the absolute and relative IR and Raman intensity differences for the LJ 12–6 parameters obtained from TIP3P, SPC/E, and TIP3P-CHARMM, with TIP3P as the reference for all parameter choices; and absolute frequency differences and the absolute and relative IR and Raman intensity differences comparing EE-QM to PE-QM (PDF)
