# Supplementary material for: Workflow for Harmonic IR and Raman Spectra of Embedded Systems: The PE-QM Approach
Source: J Phys Chem A. 2025 Jul 18;129(30):6896–910. doi: 10.1021/acs.jpca.5c00713 (PMC12319913; doi:10.1021/acs.jpca.5c00713)
Supplement: Supplementary file 1 [file jp5c00713_si_001.pdf]

Supporting Information: Workflow for Harmonic IR and Raman Spectra of Embedded Systems: The PE-QM Approach

Jonas Vester,*,† David Carrasco-Busturia,†,‡ Kenneth Ruud,¶ Magnus Ringholm,¶ and Jógvan Magnus Haugaard Olsen*,†,¶

†*DTU Chemistry, Technical University of Denmark (DTU), DK-2800 Kongens Lyngby, Denmark*

‡*Division of Theoretical Chemistry and Biology, School of Engineering Sciences in Chemistry, Biotechnology and Health, KTH Royal Institute of Technology, SE-100 44 Stockholm, Sweden*

¶*Hylleraas Centre for Quantum Molecular Sciences, Department of Chemistry, UiT The Arctic University of Norway, N-9037 Tromsø, Norway*

E-mail: jvest@kemi.dtu.dk; jmho@kemi.dtu.dk

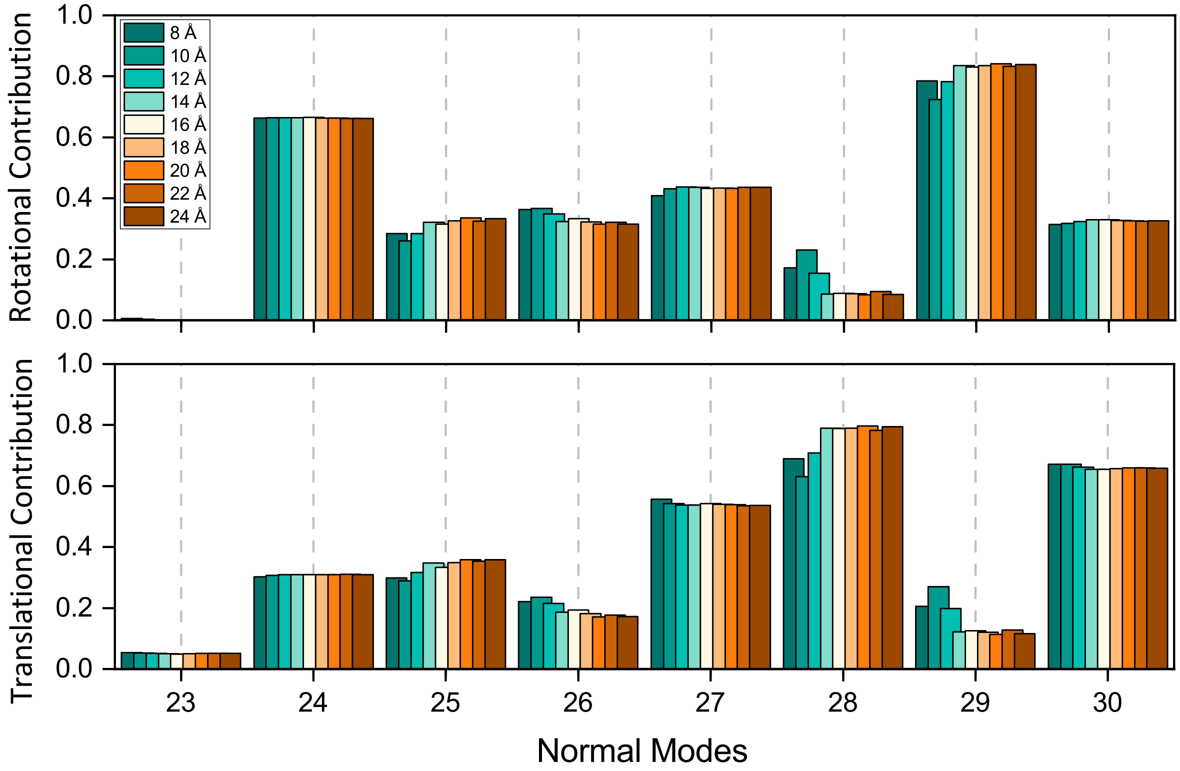

Figure S1: Pseudo-translational and pseudo-rotational contributions of the lowest frequency normal modes 23-30 of a single snapshot of acetone in water for different sizes of the molecular environment.

Table S1: Configurational Sample Size Errors for the Frequencies, IR Intensities, and Raman Intensities[a]

	Number of Snapshots[b]				
Property[c]	500	250	125	50	25
$\bar{\delta}\tilde{\nu}$	0.18 ± 0.03 (0.30)	0.28 ± 0.06 (0.46)	0.41 ± 0.10 (0.72)	0.67 ± 0.19 (1.49)	0.96 ± 0.27 (2.25)
$\bar{\delta}I_{\mathrm{IR}}$	2.05 ± 0.44 (3.34)	3.05 ± 0.99 (7.33)	4.35 ± 1.57 (10.27)	7.31 ± 2.34 (19.46)	10.25 ± 3.20 (24.10)
$\bar{\delta}_r I_{\mathrm{IR}}$	1.25 ± 0.26 (2.11)	1.91 ± 0.54 (3.52)	2.73 ± 0.81 (5.62)	4.58 ± 1.21 (9.34)	6.43 ± 1.61 (13.05)
$\bar{\delta}I_{\mathrm{Raman}}$	1.51 ± 0.43 (2.60)	2.24 ± 0.90 (5.97)	3.09 ± 1.27 (8.16)	4.73 ± 1.73 (10.87)	6.44 ± 2.37 (15.21)
$\bar{\delta}_r I_{\mathrm{Raman}}$	0.83 ± 0.22 (1.45)	1.29 ± 0.46 (3.12)	1.80 ± 0.61 (3.95)	2.75 ± 0.88 (6.10)	3.78 ± 1.18 (9.06)

[a] Entries are average absolute ($\bar{\delta}$) or relative ($\bar{\delta}_r$) window errors $\pm$ standard deviations with maximum errors in parentheses. [b] Snapshots are separated by 1.0 ps. [c] Frequency errors are in cm^{-1}, absolute IR intensity errors are in $10^{10}\cdot\mathrm{m}^2\cdot\mathrm{mol}$, absolute Raman intensity errors are in $10^{-58}\ \mathrm{C}^4\cdot\mathrm{s}^2\cdot\mathrm{m}^{-2}\cdot\mathrm{J}^{-1}\cdot\mathrm{kg}^{-1}$, and all relative values are percentages.

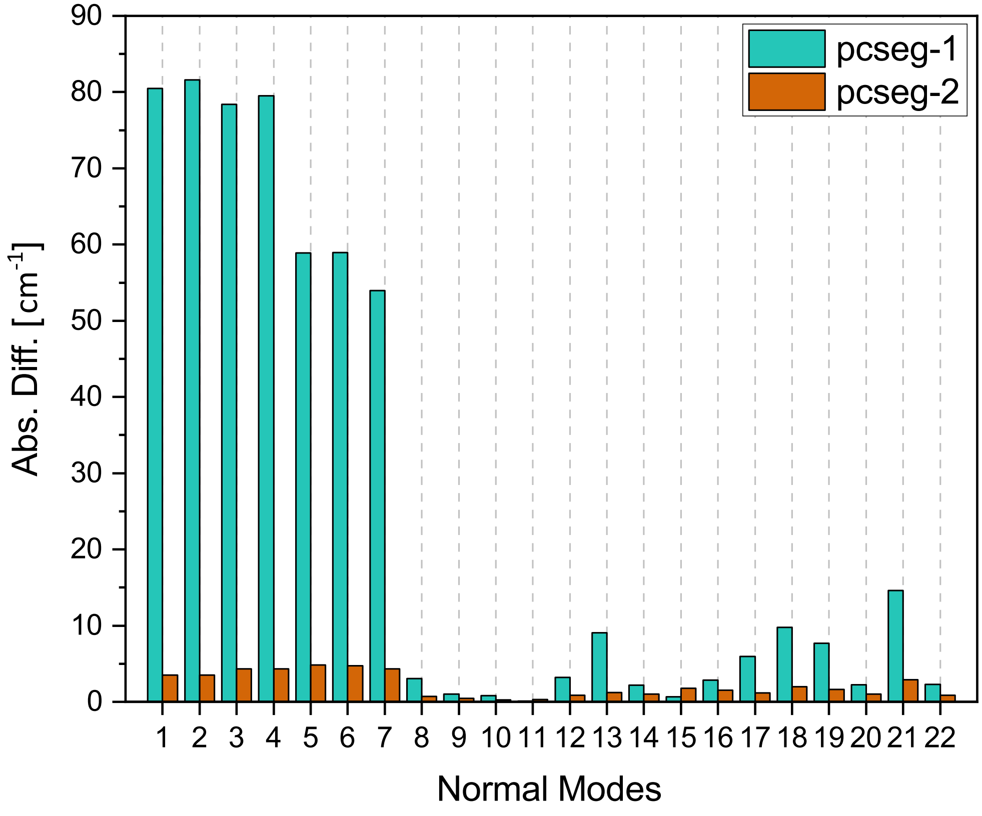

Figure S2: Convergence of vibrational frequencies with respect to basis set sizes for a single snapshot of acetone in water. Shown are absolute differences relative to reference values calculated using pcseg-3.

Table S2: Configurational Sample Size Errors for the Frequencies, IR Intensities, and Raman Intensities[a]

	Number of Snapshots[b]				
Property[c]	500	250	125	50	25
$\bar{\delta}\tilde{\nu}$	$0.15 \pm 0.02\,(0.21)$	$0.28 \pm 0.07\,(0.50)$	$0.42 \pm 0.11\,(0.71)$	$0.68 \pm 0.18\,(1.43)$	$0.99 \pm 0.29\,(2.12)$
$\bar{\delta}I_{\mathrm{IR}}$	$1.75 \pm 0.29\,(2.61)$	$2.71 \pm 0.86\,(5.61)$	$4.39 \pm 1.45\,(7.92)$	$7.39 \pm 2.17\,(13.62)$	$10.46 \pm 3.12\,(21.54)$
$\bar{\delta}_r I_{\mathrm{IR}}$	$1.05 \pm 0.22\,(1.72)$	$1.74 \pm 0.52\,(3.25)$	$2.92 \pm 0.83\,(5.92)$	$4.69 \pm 1.22\,(8.47)$	$6.65 \pm 1.73\,(13.21)$
$\bar{\delta}I_{\mathrm{Raman}}$	$1.10 \pm 0.25\,(1.75)$	$1.68 \pm 0.69\,(3.46)$	$2.86 \pm 1.17\,(6.85)$	$5.05 \pm 1.97\,(12.09)$	$7.01 \pm 2.61\,(17.74)$
$\bar{\delta}_r I_{\mathrm{Raman}}$	$0.67 \pm 0.09\,(0.91)$	$1.06 \pm 0.33\,(1.72)$	$1.69 \pm 0.55\,(3.29)$	$2.84 \pm 0.89\,(5.37)$	$4.00 \pm 1.13\,(7.92)$

[a] Entries are average absolute ($\bar{\delta}$) or relative ($\bar{\delta}_r$) window errors ± standard deviations with maximum errors in parentheses. [b] Snapshots are separated by 3.0 ps. [c] Frequency errors are in cm^{-1}, absolute IR intensity errors are in $10^{10}\cdot\mathrm{m}^2\cdot\mathrm{mol}$, absolute Raman intensity errors are in $10^{-58}\ \mathrm{C}^4\cdot\mathrm{s}^2\cdot\mathrm{m}^{-2}\cdot\mathrm{J}^{-1}\cdot\mathrm{kg}^{-1}$, and all relative values are percentages.

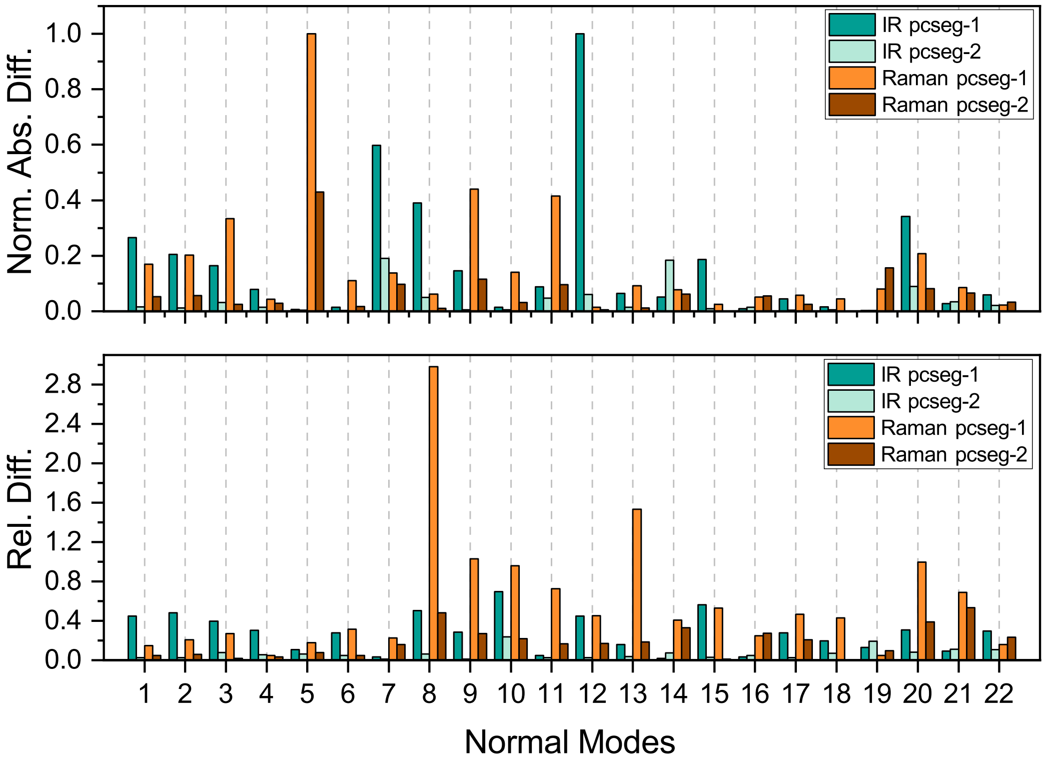

Figure S3: Convergence of IR and Raman intensities with respect to basis set size for a single snapshot of acetone in water. Shown are normalized absolute differences and relative differences compared to reference values calculated using pcseg-3. The absolute differences have been normalized to the highest absolute difference which for the IR intensities is $3.19 \cdot 10^{12}\ \mathrm{m}^2 \cdot \mathrm{mol}$ and for the Raman intensities it is $2.95 \cdot 10^{-56}\ \mathrm{C}^4 \cdot \mathrm{s}^2 \cdot \mathrm{m}^{-2} \cdot \mathrm{J}^{-1} \cdot \mathrm{kg}^{-1}$.

Table S3: Configurational Sample Size Errors for the Frequencies, IR Intensities, and Raman Intensities[a]

	Number of Snapshots[b]				
Property[c]	500	250	125	50	25
$\bar{\delta}\tilde{\nu}$	$0.17 \pm 0.04\,(0.28)$	$0.26 \pm 0.06\,(0.40)$	$0.37 \pm 0.08\,(0.68)$	$0.66 \pm 0.14\,(1.12)$	$0.97 \pm 0.24\,(1.77)$
$\bar{\delta}I_{\mathrm{IR}}$	$2.42 \pm 0.59\,(3.69)$	$3.49 \pm 1.13\,(6.53)$	$4.78 \pm 1.40\,(8.09)$	$7.07 \pm 2.01\,(15.82)$	$10.65 \pm 3.30\,(24.87)$
$\bar{\delta}_r I_{\mathrm{IR}}$	$1.26 \pm 0.20\,(1.64)$	$2.00 \pm 0.33\,(2.86)$	$2.82 \pm 0.49\,(4.20)$	$4.53 \pm 1.22\,(9.37)$	$6.74 \pm 1.91\,(14.97)$
$\bar{\delta}I_{\mathrm{Raman}}$	$1.24 \pm 0.24\,(1.72)$	$1.56 \pm 0.42\,(2.95)$	$2.69 \pm 0.75\,(5.19)$	$4.67 \pm 1.50\,(10.29)$	$6.45 \pm 2.22\,(13.94)$
$\bar{\delta}_r I_{\mathrm{Raman}}$	$0.77 \pm 0.11\,(1.02)$	$1.02 \pm 0.21\,(1.57)$	$1.65 \pm 0.36\,(2.63)$	$2.85 \pm 0.70\,(4.69)$	$3.94 \pm 1.08\,(7.29)$

[a] Entries are average absolute ($\bar{\delta}$) or relative ($\bar{\delta}_r$) window errors $\pm$ standard deviations with maximum errors in parentheses. [b] Snapshots are separated by 4.0 ps. [c] Frequency errors are in cm^{-1}, absolute IR intensity errors are in $10^{10}\cdot\mathrm{m}^2 \cdot \mathrm{mol}$, absolute Raman intensity errors are in $10^{-58}\ \mathrm{C}^4 \cdot \mathrm{s}^2 \cdot \mathrm{m}^{-2} \cdot \mathrm{J}^{-1} \cdot \mathrm{kg}^{-1}$, and all relative values are percentages.

Table S4: Configurational Sample Size Errors for the Frequencies, IR Intensities, and Raman Intensities[a]

	Number of Snapshots[b]				
Property[c]	500	250	125	50	25
$\bar{\delta}\tilde{\nu}$	$0.18 \pm 0.03\,(0.23)$	$0.29 \pm 0.03\,(0.38)$	$0.45 \pm 0.11\,(0.76)$	$0.74 \pm 0.17\,(1.28)$	$1.03 \pm 0.25\,(1.96)$
$\bar{\delta}I_{\mathrm{IR}}$	$1.65 \pm 0.39\,(2.44)$	$3.14 \pm 0.55\,(4.32)$	$4.41 \pm 0.99\,(7.11)$	$6.94 \pm 1.90\,(12.92)$	$9.97 \pm 3.04\,(22.72)$
$\bar{\delta}_r I_{\mathrm{IR}}$	$1.03 \pm 0.23\,(1.51)$	$1.98 \pm 0.39\,(3.01)$	$2.92 \pm 0.83\,(5.44)$	$4.63 \pm 1.12\,(7.69)$	$6.52 \pm 1.61\,(12.20)$
$\bar{\delta}I_{\mathrm{Raman}}$	$1.16 \pm 0.43\,(2.20)$	$1.95 \pm 0.72\,(3.41)$	$3.15 \pm 0.66\,(4.68)$	$5.08 \pm 1.81\,(11.56)$	$6.68 \pm 2.62\,(14.41)$
$\bar{\delta}_r I_{\mathrm{Raman}}$	$0.67 \pm 0.25\,(1.28)$	$1.14 \pm 0.27\,(1.69)$	$1.92 \pm 0.42\,(3.07)$	$3.03 \pm 0.81\,(5.99)$	$4.01 \pm 1.30\,(7.34)$

[a] Entries are average absolute ($\bar{\delta}$) or relative ($\bar{\delta}_r$) window errors ± standard deviations with maximum errors in parentheses. [b] Snapshots are separated by 5.0 ps. [c] Frequency errors are in cm^{-1}, absolute IR intensity errors are in $10^{10}\cdot\mathrm{m}^2\cdot\mathrm{mol}$, absolute Raman intensity errors are in $10^{-58}\ \mathrm{C}^4\cdot\mathrm{s}^2\cdot\mathrm{m}^{-2}\cdot\mathrm{J}^{-1}\cdot\mathrm{kg}^{-1}$, and all relative values are percentages.

Table S5: Absolute frequency differences, along with normalized absolute and relative differences in IR and Raman intensities using EE-LJ(TIP3P), calculated with PE-LJ(TIP3P) as the reference. The prefixes PE and EE are used to indicate that polarizable embedding or electrostatic embedding for the environment description was used.

	$\tilde{\nu}$	ε		σ'	
Normal Mode	Abs. Diff.[a]	Norm. Abs. Diff.	Rel. Diff.[b]	Norm. Abs. Diff.	Rel. Diff.
1	5.20	0.01	0.17	0.15	0.10
2	1.51	0.01	0.05	0.12	0.08
3	0.23	0.01	0.17	0.15	0.08
4	0.89	0.01	0.24	0.36	0.25
5	0.42	0.04	0.64	0.20	0.03
6	3.96	0.04	0.64	1.00	0.40
7	22.67	1.00	0.19	0.15	0.17
8	2.98	0.01	0.03	0.09	0.45
9	2.30	0.01	0.03	0.04	0.06
10	0.83	0.08	0.39	0.03	0.06
11	1.60	0.14	0.33	0.16	0.20
12	3.19	0.16	0.09	0.10	0.45
13	1.95	0.08	0.14	0.10	0.34
14	9.40	0.02	0.02	0.02	0.06
15	1.13	0.07	0.32	0.03	0.40
16	1.78	0.02	0.61	0.12	0.24
17	15.80	0.00	0.02	0.02	0.19
18	4.46	0.01	0.38	0.10	0.36
19	6.40	0.00	0.01	0.09	0.05
20	16.15	0.10	0.23	0.12	0.22
21	7.46	0.00	0.23	0.03	0.15
22	9.75	0.02	0.30	0.04	0.17

[a] Absolute frequency difference in cm^{-1}.[b] All relative values are percentages.

Table S6: Absolute frequency differences, along with normalized absolute and relative differences in IR and Raman intensities using PE-LJ(SPC/E), calculated with PE-LJ(TIP3P) as the reference. The prefix PE is used to indicate that polarizable embedding for the environment description was used.

	$\tilde{\nu}$	ε		σ'	
Normal Mode	Abs. Diff.[a]	Norm. Abs. Diff.	Rel. Diff.[b]	Norm. Abs. Diff.	Rel. Diff.
1	0.29	0.19	0.08	0.05	0.00
2	0.06	0.38	0.08	0.05	0.00
3	0.33	0.03	0.01	0.04	0.00
4	0.02	0.13	0.09	0.08	0.01
5	0.42	0.03	0.01	0.94	0.02
6	0.19	0.08	0.04	1.00	0.05
7	0.37	0.67	0.00	0.02	0.00
8	0.17	0.26	0.02	0.01	0.01
9	0.34	1.00	0.07	0.04	0.01
10	0.45	0.43	0.06	0.01	0.00
11	0.23	0.30	0.02	0.18	0.03
12	0.70	0.24	0.00	0.17	0.10
13	0.60	0.08	0.00	0.03	0.01
14	0.40	0.35	0.01	0.02	0.01
15	0.09	0.21	0.03	0.01	0.02
16	0.07	0.02	0.01	0.02	0.00
17	1.68	0.02	0.01	0.00	0.00
18	0.12	0.06	0.09	0.02	0.01
19	0.48	0.01	0.01	0.08	0.01
20	1.02	0.18	0.01	0.04	0.01
21	0.82	0.01	0.01	0.00	0.00
22	0.68	0.04	0.02	0.01	0.01

[a] Absolute frequency difference in cm^{-1}.[b] All relative values are percentages.

Table S7: Absolute frequency differences, along with normalized absolute and relative differences in IR and Raman intensities using PE-LJ(TIP3P-CHARMM), calculated with PE-LJ(TIP3P) as the reference. The prefix PE is used to indicate that polarizable embedding for the environment description was used.

	$\tilde{\nu}$	ε		σ'	
Normal Mode	Abs. Diff.[a]	Norm. Abs. Diff.	Rel. Diff.[b]	Norm. Abs. Diff.	Rel. Diff.
1	0.49	0.10	0.04	0.26	0.01
2	0.19	0.20	0.04	0.43	0.02
3	0.57	0.02	0.01	0.28	0.01
4	0.14	0.01	0.01	0.75	0.03
5	0.51	0.07	0.03	0.83	0.01
6	0.34	0.10	0.05	1.00	0.02
7	0.33	0.28	0.00	0.01	0.00
8	0.76	0.03	0.00	0.08	0.02
9	0.69	1.00	0.07	0.27	0.02
10	0.45	0.42	0.06	0.11	0.01
11	0.32	0.66	0.04	0.24	0.02
12	0.39	1.00	0.02	0.02	0.01
13	0.47	0.67	0.03	0.02	0.00
14	0.23	0.14	0.00	0.02	0.00
15	0.66	0.09	0.01	0.04	0.03
16	0.21	0.01	0.00	0.03	0.00
17	0.12	0.01	0.00	0.00	0.00
18	0.21	0.02	0.03	0.29	0.06
19	0.59	0.02	0.03	0.36	0.01
20	1.29	0.01	0.00	0.02	0.00
21	0.71	0.02	0.02	0.03	0.01
22	0.15	0.04	0.01	0.05	0.01

[a] Absolute frequency difference in cm^{-1}.[b] All relative values are percentages.
